# Incursion Preparedness, Citizen Science and Early Detection of Invasive Insects: The Case of *Aleurocanthus spiniferus* (Hemiptera, Aleyrodidae) in France

**DOI:** 10.3390/insects14120916

**Published:** 2023-11-30

**Authors:** Jean-Claude Streito, Emilie Mendes, Emmanuel Sanquer, Martin Strugarek, David Ouvrard, Victor Robin-Havret, Laurent Poncet, Christian Lannou, Jean-Pierre Rossi

**Affiliations:** 1 CBGP (Centre de Biologie pour la Gestion des Populations), INRAE, CIRAD, IRD, Institut Agro, 755 Avenue du Campus Agropolis, CS 30016, 34988 Montferrier-sur-Lez, France; jean-claude.streito@inrae.fr; 2 Independent Researcher; 3 DRAAF Occitanie (Direction Régionale de l’Alimentation de l’Agriculture et de la Forêt), 697 Avenue Etienne Méhul, CEDEX 03, 34078 Montpellier, France; emmanuel.sanquer@agriculture.gouv.fr (E.S.);; 4 ANSES, Plant Health Laboratory, Entomology and Botany Unit, 755 Avenue du Campus Agropolis, CS 30016, 34988 Montferrier-sur-Lez, France; 5 PatriNat (OFB, MNHN, CNRS, IRD), CEDEX 05, 75005 Paris, France; victor.robin-havret@mnhn.fr (V.R.-H.); laurent.poncet@mnhn.fr (L.P.); 6 INRAE, Direction Scientifique Agriculture, 147 Rue de l’Université, CEDEX 07, 15159 Paris, France; christian.lannou@inrae.fr

**Keywords:** whiteflies, early warning, biological invasions, pest insects

## Abstract

**Simple Summary:**

Invasive alien species are a major element of global change and represent a threat to global biodiversity and ecosystem integrity. Early detection of invasive species is of capital importance for management success. We describe the process by which the invasive whitefly, *Aleurocanthus spiniferus *(Hemiptera, Aleyrodidae), listed as a European quarantine organism, was detected in France. The first observation was made by a volunteer who reported a picture of an adult *Aleurocanthus spiniferus* on the Inventaire National du Patrimoine Naturel (INPN Espèces), a citizen science resource developed by l’Office Français de la Biodiversité and the French Muséum National d’Histoire Naturelle. We outline the sequence of actions that led to the official reporting of this species in France and show how incursion preparedness contributed to a rapid response. This case exemplifies how citizen science can contribute to the early detection of invasive species and highlights the importance of informing both the general public and professionals about major environmental issues.

**Abstract:**

We describe the process by which the quarantine whitefly, *Aleurocanthus spiniferus *(Hemiptera, Aleyrodidae), was detected in France. The initial observation was made by a volunteer who reported a picture of an adult in the Inventaire National du Patrimoine Naturel (INPN Espèces), a citizen science resource developed by l’Office Français de la Biodiversité and the French Muséum National d’Histoire Naturelle. The specimen was suspected to be *A. spiniferus* from this picture by one of the expert entomologists in charge of the Hemiptera group validation. Once the species was identified, it was mounted on a slide and the information was officially passed on to the ministry in charge of agriculture via a communication channel set up in advance for this type of situation. The ministry then triggered the regulatory actions planned in the event of the suspected detection of quarantine organisms. Sampling was quickly carried out and the specimens collected on this occasion were formally identified as belonging to the species *A. spiniferus*. This led to the formalization of an outbreak in France. This sequence of decisions took just two months from the first observation to the implementation of a management plan. This case presents how incursion preparedness contributes to a rapid response. Furthermore, this case exemplifies how citizen science can contribute to the early detection of invasive species and highlights the importance of informing both the general public and professionals about major environmental issues.

## 1. Introduction

Invasive alien species (IAS) are a major element of global change and represent a threat to global biodiversity and ecosystem integrity [1,2]. They have a high economic impact through direct loss of crops and biodiversity, as well as via management interventions [3], and the associated cost has strongly increased in past decades [4]. The old saying that “prevention is better than cure” obviously applies to IAS introduction as the earlier a species is detected, the better the chances to control and possibly eradicate it [5]. As the time lags between the arrival of an IAS and the start of the control campaign are determinant, surveillance and early detection have become of capital importance [5].

Epidemiological surveillance relies on both general and specific surveillance. Specific surveillance focuses on species deemed to have high invasion capability and/or introduction and establishment likelihood and which are considered to have important potential ecological, economic, or social impacts [6]. These species are monitored using very different tools, including the traditional human-conducted active means (visual and chemical methods) as well as more recent methods based on remote sensing [7]. Public participation is another fast-developing aspect of invasive species management [8] and crowd surveillance has recently emerged as another source of consistent information regarding IAS [9]. In recent years, citizen science significantly contributed to monitoring the expansion of different alien insect pests such as the oak processionary moth *Thaumetopoea processionea* [10] or the brown marmorated stinkbug *Halyomorpha halys* [11]. They are at the center of innovative projects such as Alientoma, an internet site offering a dynamic checklist and database of alien insect species in Greece [12].

However, monitoring the expansion of an invasive species is one thing, and discovering new species is another. Large, charismatic, or colorful species can be successfully detected via citizen science [9]. From this point of view, the discovery of the black shield wasp *Vespa bicolor* (Hymenoptera, Vespidae) in Spain in 2013 is a textbook case. Formal identification was carried out by an entomologist [13] from photographs taken by a citizen contributor and posted on his website [14]. The first report of *Brachyplatys subaeneus* (Hemiptera, Plataspidae) in the United States, triggered by the publication of a photograph on the website iNaturalist [15], is another example. These cases show how an insect with attractive colors and/or large size can be reported via citizen contribution. The matter is more difficult for small, inconspicuous, or hitherto undescribed species that receive less attention from citizens [9]. We will subsequently see the case of a small species without particular coloring.

Once the exotic species has been formally identified by competent scientists, the process continues with official reporting to the relevant authorities. This transfer of information is very important because it conditions the triggering of management operations. It is therefore crucial that the time elapsed between observation in the field and reporting to decision makers is as short as possible. The existence of contingency plans that set out the actions to be taken is essential for guaranteeing the maximum effectiveness of eradication or containment plans [16]. These plans define the management measures to be taken, their funding, and which stakeholders should be involved.

The present communication aims to illustrate the actors involved and the chronology of events leading to the triggering of the management plan of *Aleurocanthus spiniferus* in mainland France in June 2023.

## 2. *Aleurocanthus spiniferus*, the Citrus Spiny Whitefly

*Aleurocanthus spiniferus* (Quaintance, 1903) belongs to the whitefly family (Hemiptera, Sternorrhyncha, Aleyrodidae), a group of minute phytophagous hemipterans that comprises more than 1610 described species [17,18]. The genus *Aleurocanthus* Quaintance & Baker, 1914, comprises 91 species, while many more probably remain undescribed. The descriptions of many species are inconsistent and the genus *Aleurocanthus* would undoubtedly benefit from complete revision.

**Figure 1:**
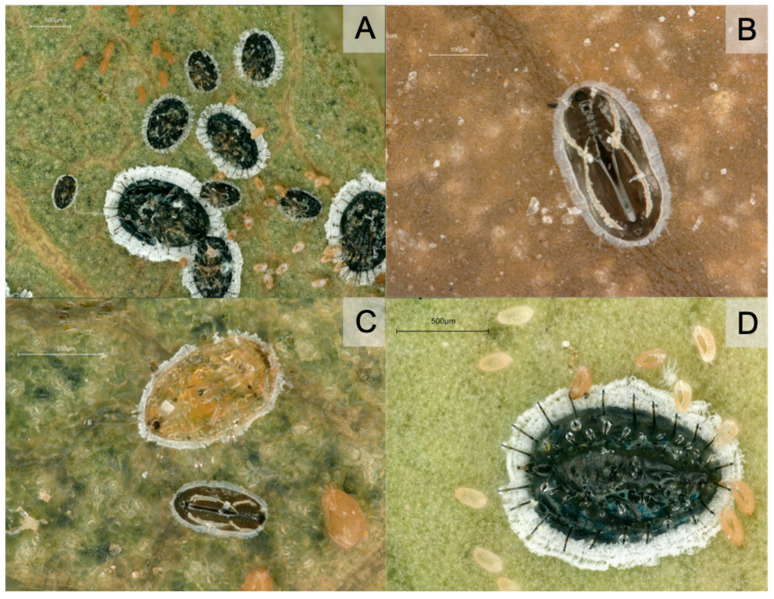
*Aleurocanthus spiniferus* from French outbreak: (**A**), larvae and puparia; (**B**), first immature stage; (**C**), first and second immature stages; (**D**), puparium (last immature stage) and eggs. (**A**–**D**) photo J.-C.S.

**Figure 2:**
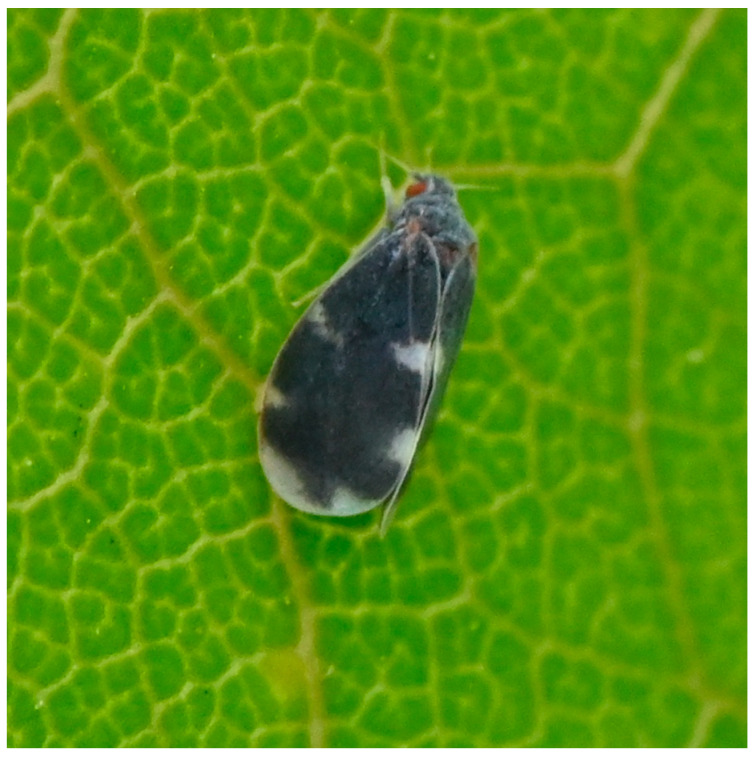
*Aleurocanthus spiniferus*, adult. Photo E.M.

International regulations on plant protection focus on three species of *Aleurocanthus* that are agricultural pests of importance: *A. citriperdus* Quaintance & Baker, 1916, *A. woglumi* Ashby, 1915, and *A. spiniferus*. These species are all pests of *Citrus* trees among other crops. These species can be recognized in the field by their last immature stage (called puparium). These are dark brown to black, with a short fringe of white wax and the presence of glandular spines on the upper side (Figure 1). Adults have blue-grey wings with white markings (Figure 2), while many other whiteflies are mostly white (as pointed out by the usual name given to this family). As a consequence, the identification of the group formed by *A. citriperdus*, *A. spiniferus*, and *A. woglumi* is quite easy, even on pictures posted on websites. On the other hand, distinguishing between species is more difficult and requires slide mounting, microscopic observation by a specialist, consultation of reference collections, and specialized literature. The EPPO Standard (2022) was written to help national laboratories in charge of quarantine pest identification to identify these species [19]. The EPPO standard also recommends that molecular identification be used cautiously given the unreliability of sequences currently available in international databases [19].

*A. spiniferus* is amongst the most damaging *Aleurocanthus* species worldwide. It is highly polyphagous as more than 100 host plants from 37 families have been recorded [20,21]. Important crops under threat include *Citrus* spp., *Vitis vinifera*, and *Rosa* spp. The species is present on the European Union quarantine list (Part B Annex II, quarantine pests known to occur in the union territory of the regulation (EU) 2019/2072) and on the EPPO A2 list (pests recommended for regulation as quarantine pests locally present in the EPPO region). *A. spiniferus* is a successful invasive species that originates from tropical Asia and has spread across the Pacific, to Central, Eastern, and Southern Africa, via the Indian Ocean, and more recently across Europe, where it was first reported from Italy (2008) [22], then from Croatia (2012) [23], Montenegro (2013) [24], Greece (2016) [25] and Albania (2018) [26]. It was not reported from France until 2023.

## 3. Chronology of Events

One of the authors (EM) observed an unknown insect in her garden (Bernis, Gard department, France), took several pictures (Figure 2), and identified the specimen as a species belonging to the genus *Aleurocanthus*. She posted the pictures (17 April 2023 17:25 Emi_ms # ID data: 648216) on the INPN espèces web site developed by the Muséum National d’Histoire Naturelle (MNHN). INPN stands for Inventaire National du Patrimoine Naturel (National Inventory of Natural Heritage). It is a gateway for biodiversity and geodiversity in France and publishes knowledge on animal, plant, and fungi species, natural environments, protected areas, and geological heritage (https://inpn.mnhn.fr/accueil/participer/inpn-especes accessed on 24 November 2023). It harbors datasets validated by networks of experts and is available to all, whether they are professionals, amateurs, or citizens. INPN proposes a citizen science resource that allows citizens to post pictures along with some optional details such as observation location. Once the pictures are posted, they are initially checked and sorted by taxonomic group, then they become available to the expert scientists in charge of validating the sightings.

One of the authors, JCS, who is one of the experts in charge of the Hemiptera, observed the photograph and found that the identification of the genus of the specimen proposed by the contributor was correct. Knowing that this genus contains several regulated pest species, he contacted the INPN website administrators to get in touch with the person (the anonymous observer) who had filed the report to obtain further information. In particular, he sought to check the accuracy of the date and the geographical location of the report (6 May 2023).

The observer (EM) agreed to be contacted on 23 May 2023 and provided additional photographs, confirmed the location and date of the sighting, and indicated that she did not observe any immature stages. This information was sufficient, given the genus in question, to alert the French NPPO (National Plant Protection Organization) at the ministry in charge of agriculture (DGAL, Direction Générale de l’Alimentation). As the alert was stemming from INRAE (Institut National de Recherche pour l’Agriculture, l’Alimentation et l’Environnement) scientists, it was agreed to use the INRAE reporting procedure, and one of the authors, JPR, reported the sighting to the ministry on the 26 May 2023. Meanwhile, EM returned to the site of the initial sighting and observed numerous larvae and adults. On 7 June 2023, the ministry contacted EM and JCS. They then sent phytosanitary inspectors to the site, who confirmed the outbreak and collected samples on 15 and 16 June 2023. Specimens were subsequently sent to the LSV (Laboratoire de la Santé des Végétaux/Plant Health Laboratory), which is part of ANSES (Agence nationale de sécurité sanitaire de l’alimentation, de l’environnement et du travail/French Agency for Food, Environmental and Occupational Health & Safety). ANSES is the institution officially responsible for identifying quarantine organisms in France. DO received the samples on 19 June 2023 and confirmed that the specimens were indeed individuals of *Aleurocanthus spiniferus* on 20 June 2023, thus making the species officially present in France. In parallel, several authorities and directorates including the ministry were alerted through the internal reporting system in ANSES called ‘SALSA’ (for ‘Système d’ALertes Sanitaires de l’Anses’).

The morphological identification of *A. spiniferus* was based on slide-mounted puparia. *A. spiniferus* was distinguished from close species following the EPPO diagnostic standard [19]. We additionally compared the specimens with the slides of Aleyrodidae species from the INRAE-CBGP continental Arthropod Collection (https://doi.org/10.15454/D6XAKL accessed on 24 November 2023).

Following this detection, the ministry immediately triggered a contingency plan and launched management operations planned for that situation. These actions included (1) investigating the possible sources of introduction, (2) assessing the actual distribution of the pest using a delimiting survey, (3) consignment and sanitization of plants intended for circulation to prevent further spread of the pest until the appropriate and mandatory phytosanitary measures are applied by professional operators, (4) quantifying unreported damages to crops and other plant species in the area, and (5) raising the awareness of stakeholders (using targeted messages using existing communication channels such as “Bulletin de santé du végétal”) as well as of citizens (through a regional press release on 21 July 2023).

Interestingly, the public information campaign launched by the ministry triggered dozens of citizen sightings in the area. Some observations occurred before the initial sighting that led to the sequence of events reported here. Some citizens were not aware of the existence of the INPN resource or did not suspect they had observed a regulated pest. As of today, the oldest confirmed photograph of *Aleurocanthus spiniferus* taken in the area dates back to December 2020, which is more than 2 years before the observation reported in the present communication.

## 4. Discussion

Roughly two months separated the initial observation, filed in the participatory science system, and the official recognition of the presence of *Aleurocanthus spiniferus* in mainland France. This process was quick, especially if we consider the unexpected nature of the discovery and the number of protagonists implied at different stages of the process. This report firstly benefited from the gateway system for biodiversity and geodiversity, which was developed and maintained by the French National Museum of Natural History and by the French Office for Biodiversity. This structure has good visibility in France, which plays an important role in the present case. For example, the platform collected 186,946 reports from 2884 contributors in 2022. The museum’s services are responsive and, once the photo was recognized as potentially representing a regulated agricultural pest, the exchanges were very fluid.

In recent years, INRAE has set up an internal procedure to centralize the reporting of problematic species by its agents. Sightings are checked within the institute. If they correspond to regulated species, they are rapidly reported to the ministry in charge of agriculture, which is the final decision maker. As a result, three days passed between discussions with the citizen and the report to the ministry. The rest of the chain also functioned well. Official services quickly and efficiently organized field sampling and sent specimens to the service responsible for official expertise (ANSES). It only took ANSES 1 day to formulate official diagnoses. The rapid identification of *A. spiniferus* greatly benefited from prior anticipation of the difficulties inherent in identifying species of the genus *Aleurocanthus* by two of the authors (DO, JCS), who wrote the EPPO standard guiding the identification of *A. citriperdus*, *A. spiniferus* and *A. woglumi* [19].

Even if the case of the reporting of *Aleurocanthus spiniferus* in France is an example of a successful early warning, the system based on citizen science can certainly be improved. First of all, it must be emphasized that the identification of sightings posted by volunteers is carried out by experts for whom this is not their primary mission. For example, here JCS is an INRAE agent acting as an INPN Espèces expert voluntarily. This is often the case for certain groups such as Diptera. The same holds for the other mobilized experts. As a consequence, some photos may not be examined within a time frame compatible with an early warning.

It should also be noted that the number of experts varies with the taxonomic groups concerned, which means that in certain cases (e.g., Aphididae and Coccomorpha) the detection of quarantine species could take a long time or even not be carried out. In the case of whiteflies, difficulties include the small size of the species and the fact that accurate species identification requires the slide-mounting of specimens [27]. This is also the case for most Hemiptera Sternorrhyncha. The genus *Aleurocanthus* is an exception within this group, with adults recognizable in photos by a specialist.

In the longer term, and in a very general way, we must emphasize the importance of the role of taxonomists in the identification of regulated organisms on the basis of morphological characters, because it is on this basis that a species is first detected and usually officially declared present in a country. The number of specialists must therefore be maintained, and the discipline must remain alive in the academic world, if we want to maintain these skills. Since the initial identification of the sequenced specimen is correct, DNA barcoding is an efficient way to assign unidentified specimens to known species but the number of errors should not be underestimated [28,29]. Deep learning technologies constitute interesting ways to implement automatic species recognition from pictures [30]. They could be used to carry out an initial sorting of photographs hosted on the citizen science database [31] and lighten the taxonomists’ workload.

The small size of *A. spiniferus* means that it attracts little attention from non-specialist person unless it is associated with visible damage to plants. However, the sequence of events reported here shows that an earlier detection could have been obtained if the observers had disseminated the observations to competent biologists, such as through a citizen science resource. This point is important because it emphasizes the importance that must be given to public awareness and education on environmental issues and in particular biological invasions. It is through this means that citizens feel concerned and become involved in observing biodiversity and reporting the species encountered.

## Data Availability

The data presented in this study (High-definition pictures of different stages of *Aleurocanthus spiniferus*) are openly available from a public repository accessible at https://doi.org/10.57745/WOHBMY accessed on 24 November 2023.
